# Growth and Antioxidant Responses in Iron-Biofortified Lentil under Cadmium Stress

**DOI:** 10.3390/toxics9080182

**Published:** 2021-07-31

**Authors:** Ruchi Bansal, Swati Priya, Harsh Kumar Dikshit, Sherry Rachel Jacob, Mahesh Rao, Ram Swaroop Bana, Jyoti Kumari, Kuldeep Tripathi, Ashok Kumar, Shiv Kumar, Kadambot H. M. Siddique

**Affiliations:** 1 Division of Germplasm Evaluation, ICAR—National Bureau of Plant Genetic Resources, New Delhi 110012, India; swatipriya9013@gmail.com (S.P.); Jyoti.Kumari@icar.gov.in (J.K.); Kuldeep.Tripathi@icar.gov.in (K.T.); Ashok.Kumar28@icar.gov.in (A.K.); 2 Division of Genetics, ICAR—Indian Agricultural Research Institute, New Delhi 110012, India; 3 Division of Germplasm Conservation, ICAR—National Bureau of Plant Genetic Resources, New Delhi 110012, India; Sherry.Jacob@icar.gov.in; 4 ICAR—National Institute of Plant Biotechnology, New Delhi 110012, India; mraoiari@gmail.com; 5 Division of Agronomy, ICAR—Indian Agricultural Research Institute, New Delhi 110012, India; rsbana@gmail.com; 6 International Centre for Agricultural Research in Dryland Areas, Avenue Hafiane Cherkaoui, Rabat 10112, Morocco; SK.Agrawal@cgiar.org; 7 The UWA Institute of Agriculture, The University of Western Australia, Perth, WA 6001, Australia; kadambot.siddique@uwa.edu.au

**Keywords:** antioxidant, cadmium, dry weight, heavy metal, iron, lentil

## Abstract

Cadmium (Cd) is a hazardous heavy metal, toxic to our ecosystem even at low concentrations. Cd stress negatively affects plant growth and development by triggering oxidative stress. Limited information is available on the role of iron (Fe) in ameliorating Cd stress tolerance in legumes. This study assessed the effect of Cd stress in two lentil (*Lens culinaris* Medik.) varieties differing in seed Fe concentration (L4717 (Fe-biofortified) and JL3) under controlled conditions. Six biochemical traits, five growth parameters, and Cd uptake were recorded at the seedling stage (21 days after sowing) in the studied genotypes grown under controlled conditions at two levels (100 μM and 200 μM) of cadmium chloride (CdCl_2_). The studied traits revealed significant genotype, treatment, and genotype × treatment interactions. Cd-induced oxidative damage led to the accumulation of hydrogen peroxide (H_2_O_2_) and malondialdehyde in both genotypes. JL3 accumulated 77.1% more H_2_O_2_ and 75% more lipid peroxidation products than L4717 at the high Cd level. Antioxidant enzyme activities increased in response to Cd stress, with significant genotype, treatment, and genotype × treatment interactions (*p* < 0.01). L4717 had remarkably higher catalase (40.5%), peroxidase (43.9%), superoxide dismutase (31.7%), and glutathione reductase (47.3%) activities than JL3 under high Cd conditions. In addition, L4717 sustained better growth in terms of fresh weight and dry weight than JL3 under stress. JL3 exhibited high Cd uptake (14.87 mg g^−1^ fresh weight) compared to L4717 (7.32 mg g^−1^ fresh weight). The study concluded that the Fe-biofortified lentil genotype L4717 exhibited Cd tolerance by inciting an efficient antioxidative response to Cd toxicity. Further studies are required to elucidate the possibility of seed Fe content as a surrogacy trait for Cd tolerance.

## 1. Introduction

Cadmium (Cd) stress is an important issue of global concern. This hazardous heavy metal enters the environment through various industrial and agricultural anthropogenic activities. It may pollute water or soil, and its accumulation in plants and animals poses serious threats to human health [1]. Since Cd is mobile and soluble in nature, it is easily taken up by plants. Its uptake adversely affects plant growth and development and causes toxicity symptoms, including necrotic lesions, stunted growth, and chlorosis [2]. Cd inhibits photosynthesis by retarding the rate of electron transport, chlorophyll fluorescence, stomatal conductance, and enzymatic reactions in carbon fixation [3]. Severe reductions in growth and yield of plants exposed to Cd toxicity have been documented in crops such as mung bean [4], tomato [5], rice [6], and wheat [7].

Studies on Cd uptake, transport, and accumulation have shown that Cd competes with other metals at their absorption sites, leading to their substitution and causing disbalance in metabolic activities [8]. Cd, a divalent cation, competes with iron (Fe), magnesium (Mg), and calcium (Ca) during membrane transport. Fe deficiency was associated with Cd toxicity by Fe-dependent gene regulation in *Arabidopsis* [9,10]. Cd binds with the sulfhydryl groups of proteins and impairs protein function [11]. The cell’s redox state subsequently changes, producing reactive oxygen species (ROS) in plants that can damage lipids, proteins, and other macromolecules, such as DNA and RNA. Different enzymatic and non-enzymatic antioxidants detoxify ROS to cope with oxidative damage. Upregulation of antioxidant activity in response to Cd-induced toxicity has been reported in wheat, rice, chickpea, and mung bean [12,13,14,15].

To mitigate Cd stress, application of nutrients such as Fe has been suggested for rice [16], wheat [7], and mung bean [4]. Ca and Fe enhanced the activity of various antioxidant enzymes in mung bean and chickpea, respectively, to ameliorate Cd stress [4,13]. In Fe-supplemented mung bean in a Cd-rich environment, shoot biomass had strong correlations with malondialdehyde, hydrogen peroxide (H_2_O_2_), peroxidase, polyphenol oxidase, and glutathione reductase activities [4].

Studies on Cd stress responses are available primarily in cereals with the minimal information reported in legumes. Lentil (*Lens culinaris* Medik.) is an important legume crop cultivated worldwide as a source of dietary protein. Lentil has been biofortified with Fe and zinc (Zn) to address micronutrient malnutrition. While some studies are available for various legumes on improving Cd tolerance by supplementing with Fe, Zn, and Ca, no such information is available for lentil. Since Fe supplementation could improve Cd tolerance in legumes, we used Fe-biofortified lentil varieties for this study. We hypothesized that lentil varieties with high Fe content in the seed will ameliorate Cd stress. In this study, we evaluated the antioxidative and growth responses of two lentil varieties with differing seed Fe contents to Cd toxicity.

## 2. Materials and Methods

### 2.1. Experimental Material and Treatments

The experimental material comprised two lentil varieties, L4717 and JL3, released for Central India. L4717, also known as Pusa Ageti Masoor (Fe concentration 65 ppm), is a medium-sized seed, biofortified lentil variety developed at the Indian Agricultural Research Institute, New Delhi, India [17] and JL3 (Fe concentration 45 ppm) was developed at Jawaharlal Nehru Krishi Vishwavidyalaya, Jabalpur, Madhya Pradesh, India. The study was undertaken in controlled growth conditions in a polycarbonate house (28–23 °C day/night temperatures and 75% relative humidity) at the Phenotyping facility, National Institute of Plant Biotechnology, New Delhi. Seeds were treated with 1% sodium hypochlorite for 2–3 min, followed by washing with distilled water. The washed seeds were germinated on filter paper. Five-day-old seedlings were transferred in five replications to pots (8 cm diameter) containing 500 g of soil. The field topsoil (0–20 cm depth) was collected from the Research farm, Indian Agricultural Research Institute, New Delhi (28.6410° N, 77.1539° E). The soil pH was 7.1, and nitrogen, potassium, and phosphorus contents were 62 kg ha^−1^, 48 kg ha^−1^, and 155 kg ha^−1^, respectively. Seedlings were exposed to two cadmium chloride (CdCl_2_) levels (100 μM and 200 μM) based on seedling survival in a standardization experiment carried out at different Cd concentrations (50–500 μM). In the control, seedlings were irrigated with normal water as needed. Plants were harvested to record different biochemical and growth traits after 21 days of stress.

### 2.2. Determination of Biochemical Parameters

Biochemical assays (H_2_O_2_, lipid peroxidation, peroxidase, catalase, glutathione reductase, superoxide dismutase) were carried out on freshly harvested root samples.

To measure H_2_O_2_ content, 100 mg of root tissue was homogenized in 1 mL of 0.1% (*w*/*v*) trichloroacetic acid followed by centrifugation at 12,000× *g* for 15 min [18]. The extract (0.5 mL) was mixed with 0.5 mL of 10 mM potassium phosphate buffer (pH 7.4) and 1 mL of 1 M potassium iodide. Absorbance was read at 390 nm, and H_2_O_2_ content calibrated using a standard curve.

Lipid peroxidation was recorded by measuring malondialdehyde (MDA) content in the form of thiobarbituric acid-reactive substances (TBARS) [19]. A 100 mg root sample was extracted in 5% (*w*/*v*) trichloroacetic acid (TCA) and centrifuged at 10,000× *g* for 20 min. The extract (0.5 mL) was mixed with 1 mL of 0.5% (*w*/*v*) thiobarbituric acid in 20% TCA. The reaction was carried out at 95 °C for 20 min, cooled immediately, and centrifuged again at 4000× *g* for 10 min. Absorbance was read at 532 nm, and non-specific absorption was recorded at 600 nm.

Peroxidase (POX, EC 1.11.1.7) activity was recorded spectrophotometrically [20]. The enzyme was extracted from 100 mg of root tissue homogenized in 5 mL of 0.1 M phosphate buffer (pH 6.4). The reaction mixture (5 mL) contained 0.2 mL of supernatant, 50 µM pyrogallol, 50 µM H_2_O_2_, and 0.1 M phosphate buffer (pH 6.4), which was incubated at 25 °C for 5 min. To terminate the reaction, 5% H_2_SO_4_ was added, with absorbance recorded at 420 nm. Enzyme activity was represented as enzyme unit (EU) and one unit was defined as the amount of enzyme that generates 1 mol purpurogallin per min in assay conditions.

To measure catalase (CAT, EC 1.11.1.6) activity, 100 mg of root tissue was homogenized in 5 mL of 0.1 M phosphate buffer (pH 6.4) and centrifuged at 10,000× *g* at 4 °C for 20 min using a refrigerated centrifuge. The reaction mixture comprised 2.6 mL of 0.1 M phosphate buffer (pH 6.4), 0.2 mL of supernatant, and 0.1 mL of 10 mM H_2_O_2_ [21]. Changes in absorbance at 230 nm were recorded using a microplate spectrophotometer. Enzyme activity was shown as EU calculated using 36 mM cm^−1^ as the extinction coefficient

Superoxide dismutase (SOD, EC1.15.1.1) activity was measured spectrophotometrically [22]. The enzyme was extracted by homogenizing 100 mg of root tissue in 5 mL of 0.1 M phosphate buffer (pH 7.5). The reaction mixture (3 mL) contained 1.5 M sodium carbonate, 200 mM methionine, 2.25 mM NBT, 0.3 mM EDTA, 100 mM potassium phosphate buffer, and 0.1 mL of enzyme extract. To start the reaction, 0.1 mL of riboflavin (60 µM) was added, and absorbance was read at 560 nm in a spectrophotometer. One EU was defined as 50% inhibition of basic rate of reaction.

To measure glutathione peroxidase (GR, EC 1.6.4.2) activity, 100 mg of root tissue was extracted in 5 mL of 0.5 M potassium phosphate buffer (pH 7). The reaction was carried out with 50 mM potassium phosphate buffer (pH 7), containing 10% (*w*/*v*) polyvinylpyrrolidone (PVP), 0.25% (*v*/*v*) Triton X-100, 1 mM phenylmethylsulfonyl fluoride, 1 mM dehydroascorbate reductase (DHAR, EC 1.8.5.1), and 1 mM monodehydroascorbate reductase (MDHAR, EC 1.6.5.4) [23]. Absorbance was read at 340 nm. The reduction in absorbance due to oxidation of NADPH into NADP during the reaction indicated enzyme activity shown as EU.

### 2.3. Determination of Growth Parameters and Cd Content

Total biomass (fresh weight, FW and dry weight, DW), total length (TL), shoot length (SL), and root length (RL) were recorded after harvesting 21-day-old seedlings. TL, SL, and RL were measured using a long ruler. FW was recorded immediately after harvest, and DW was recorded for all samples after oven drying at 65 °C for 48 h. To measure Cd content, dried plants were ground and digested using concentrated nitric acid [24]. Cd content was quantified using an atomic absorption spectrometer.

### 2.4. Statistical Analysis

Data analysis was carried out using SPSS version 16.0 with five replicates for each treatment. Significant differences among all factors were measured using analysis of variance (ANOVA) and the least significant difference. Significant differences were estimated at *p* < 0.05 and *p* < 0.01. Pearson’s correlation coefficients were calculated using mean values of both the genotypes for the recorded traits under stress conditions.

## 3. Results

The ANOVA revealed that the recorded biochemical and growth parameters significantly differed for treatments, genotypes, and their interaction.

### 3.1. Effect of Cd on Biochemical Parameters

**Figure 1:**
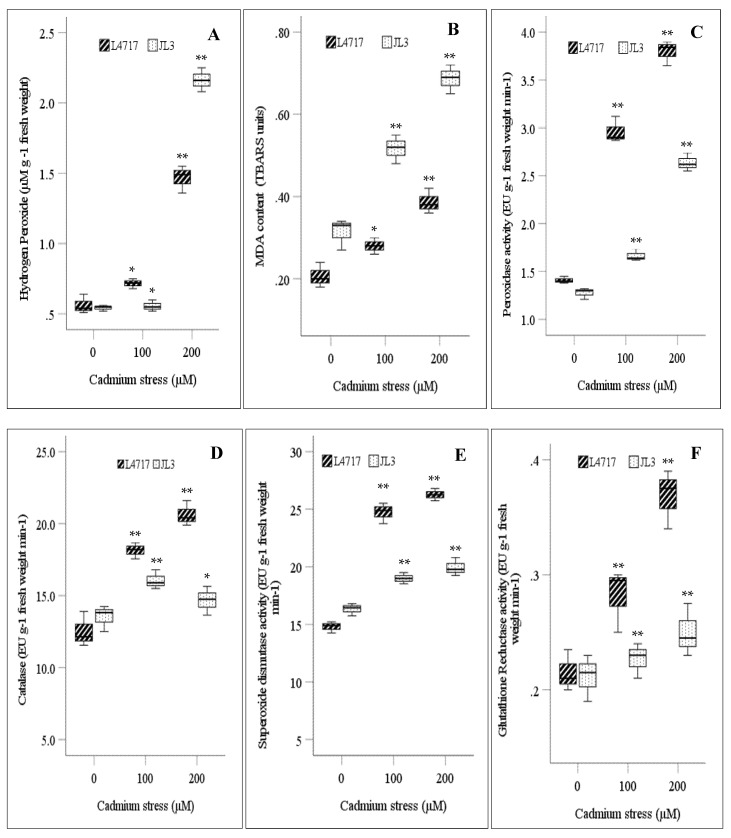
Changes in (**A**) hydrogen peroxide, (**B**) malondialdehyde, (**C**) peroxidase, (**D**) catalase, (**E**) superoxide dismutase, and (**F**) glutathione reductase activity in lentil seedlings under Cd stress. The central bar in each box represents the median, and each box shows the interquartile range. * and ** indicate significant differences among treatments at *p* < 0.05 and *p* < 0.01, respectively.

Increasing Cd concentration increased H_2_O_2_ content and lipid peroxidation in both lentil genotypes, indicating stress-mediated oxidative injury (Figure 1A,B). At 100 µM, L4717 accumulated 27.2% more H_2_O_2_ than JL3, while at higher Cd (200 µM), JL3 significantly increased H_2_O_2_ content; genotype, treatment, and interaction effects were significant at *p* < 0.01 (Table 1).

JL3 had significantly higher MDA contents than L4717 in both Cd treatments (Figure 1B). At 100 µM Cd, MDA content increased by 64.9% in JL3 and 35.5% in L4717, relative to the controls. At 200 μM Cd, MDA content increased by 87% in L4717 and 119% in JL3, relative to the controls. The F values revealed significant differences for treatment, genotype, and their interaction (*p* < 0.01) (Table 1).

Significant differences (*p* < 0.01) occurred for treatment, genotype, and their interaction for all measured enzymes (POX, CAT, SOD, and GR) (Table 1). Mean POX activity increased by 72.3% at 100 μM Cd and 139.5% at 200 μM Cd. POX activity in L4717 increased by more than two and three times at 100 µM and 200 µM Cd, respectively, relative to the controls (Figure 1C).

CAT activity also increased significantly in response to Cd stress (Figure 1D), more so in L4717 than JL3. CAT activity increased by 44.7% in L4717 and 18.8% in JL3 at 100 μM Cd. At 200 μM Cd, CAT activity increased by 64.6% in L4717 but decreased slightly in JL3.

L4717 increased SOD activity more than JL3 in both Cd treatments (Figure 1E). No significant differences in SOD activity occurred in JL3 at 100 µM or 200 µM Cd, relative to the control. In L4717, SOD activity increased by 67.2% at 100 μM Cd and 77.6% at 200 μM Cd.

Similarly, GR activity increased by 31% in L4717 but not in JL3 at 100 μM Cd and by 71.3% in L4717 and 22.1% in JL3 at 200 µM Cd (Figure 1F).

### 3.2. Effect of Cd Stress on Seedling Growth

The measured growth parameters (TL, SL, RL, FW, DW, and Cd content) significantly decreased in response to Cd stress (Table 2).

**Figure 2:**
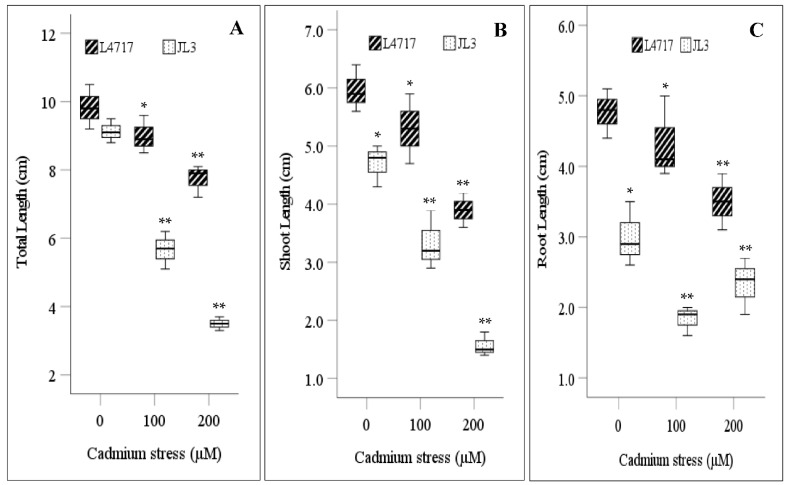

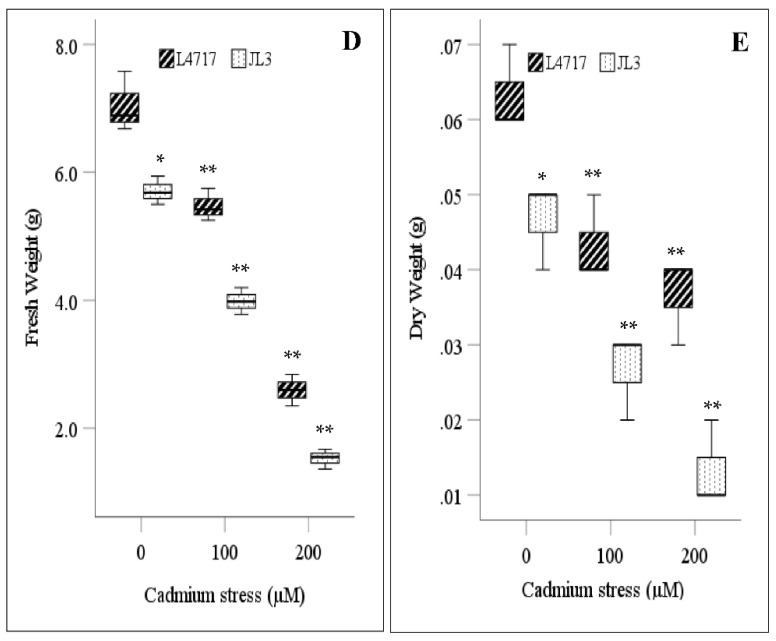
Changes in (**A**) total length, (**B**) shoot length, (**C**) root length, (**D**) fresh weight, and (**E**) dry weight of lentil seedlings under Cd stress. The central bar in each box represents the median, and each box shows the interquartile range. * and ** indicate significant differences among treatments at *p* < 0.05 and *p* < 0.01, respectively.

The genotypic mean for TL declined by 22.7% at 100 µM Cd and 40.8% at 200 μM Cd. At 100 µM Cd, TL decreased by 8.5% in L4717 and 38% in JL3; at 200 μM Cd, TL decreased by 21.4% in L4717 and 61.7% in JL3 (Figure 2A). Genotype, treatment, and their interaction for TL differed significantly at *p* < 0.01 (Table 2).

The genotypic means for SL and RL declined by 19.1% and 2.7% at 100 μM Cd, and 48.8% and 2.7% at 200 μM Cd, respectively. Genotype and treatment differences were significant for both traits at *p* < 0.01, while their interaction was significant at *p* < 0.05 (Table 2). SL and RL declined more in JL3 than L4717 in both Cd treatments (Figure 2B,C). For L4717, RL declined more at 100 µM Cd than 200 μM Cd; for JL3, RL declined with increasing Cd level. SL decreased in both genotypes with increasing Cd level.

Total biomass (FW and DW) declined significantly in both genotypes under Cd stress (Figure 2D,E). Genotype and treatment differences were significant at *p* < 0.01, while their interaction was significant at *p* < 0.05 (Table 2). The genotypic mean for FW and DW declined by 25.8% and 37.2% at 100 μM Cd and 54.1% and 60.2% at 200 μM Cd, respectively. FW declined more in JL3 than L4717 (73.3% vs. 49.6%), relative to the controls under high Cd conditions (Figure 2D). DW declined by 36.8% in L4717 and 71.4% in JL3 at 200 µM Cd (Figure 2E).

### 3.3. Cd Uptake

**Figure 3:**
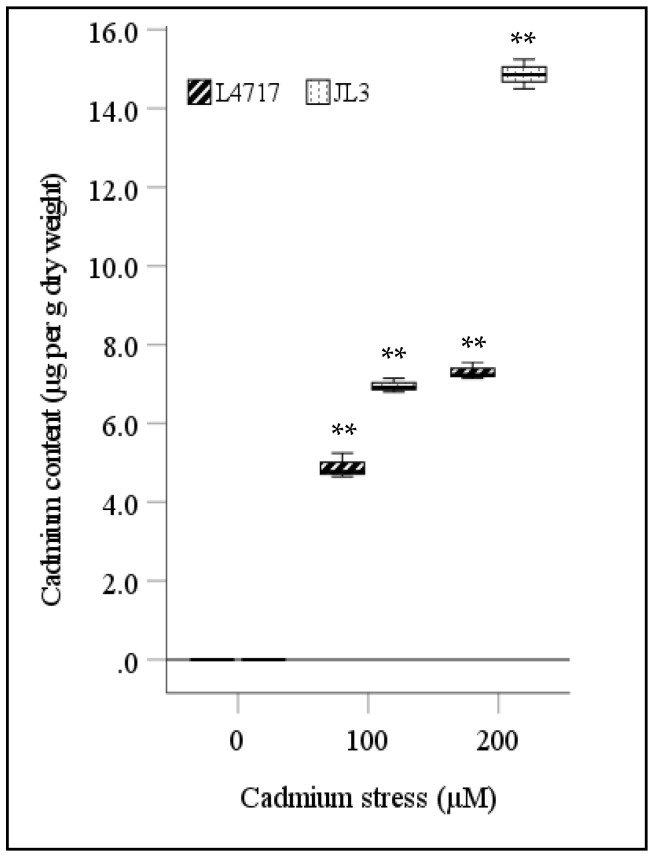
Cd uptake in lentil seedlings under Cd stress. The central bar in each box represents the median, and each box shows the interquartile range. * and ** indicate significant differences among treatments at *p* < 0.05 and *p* < 0.01, respectively.

Significant differences (*p* < 0.01) in total Cd accumulation occurred for genotype, treatment, and their interaction (Table 2). JL3 had greater Cd uptake than L4717 in both Cd treatments—42.1% higher at 100 µM Cd and 103.2% higher at 200 µM Cd (Figure 3).

### 3.4. Pearson’s Correlations Analysis to Cd Stress

**Figure 4:**
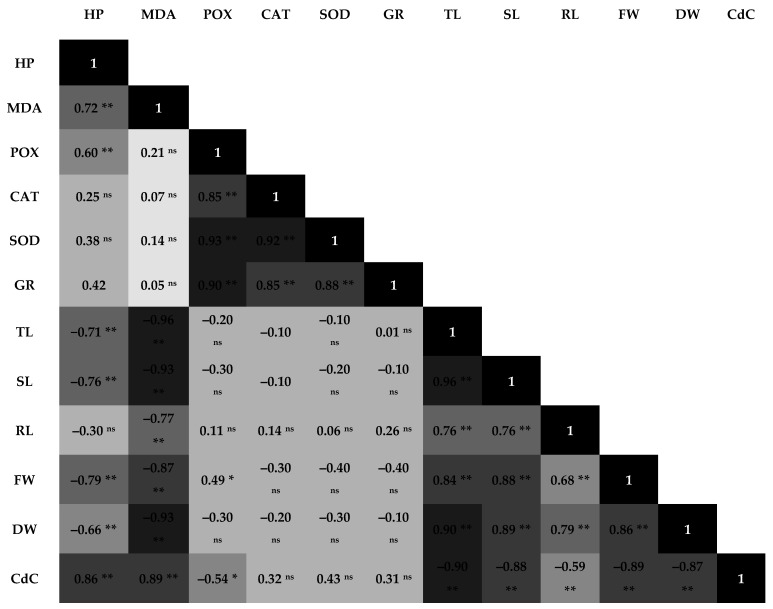
Correlation matrix of different traits under Cd-rich conditions, **^ns^** non-significant, * significant at *p* < 0.05, ** significant at *p* < 0.01. HP: hydrogen peroxide, MDA: malondialdehyde, POX: peroxidase, CAT: catalase, SOD: superoxide dismutase, GR: glutathione reductase, TL: total length, SL: shoot length, RL: root length, FW: fresh weight, DW: dry weight, CdC: Cd content. Color intensity increases with increasing correlations between traits.

Pearson’s correlations were undertaken to determine the relationship between different biochemical and growth traits in lentils under Cd stress (Figure 4). FW and DW had positive correlations with TL, SL, and RL. Various growth traits (FW, DW, TL, SL, and RL) had significant negative correlations with oxidative injuries (H_2_O_2_ and MDA contents). Significant negative correlations occurred between DW and H_2_O_2_ (r = −0.66), MDA (r = −0.93) and Cd content (r = −0.87). Cd uptake had positive correlations with H_2_O_2_ (r = 0.86) and MDA (r = 0.89) and a negative correlation with POX activity (r = −0.54). Significant positive correlations were recorded between H_2_O_2_ and MDA (r = 0.72) and H_2_O_2_ and POX activity (r = 0.60). Significant positive correlations also occurred between the activities of POX and CAT (r = 0.85), SOD and POX (r = 0.93), GR and POX (r = 0.90), and SOD and GR (r = 0.88), but enzyme activities did not significantly correlate with growth parameters.

## 4. Discussion

Studies have shown that Cd toxicity inhibits plant growth and development, adversely affecting physiological mechanisms, including germination, photosynthesis, ion transport, and mineral nutrition, resulting in reduced biomass and yield in different crops [25,26,27]. Cd is easily transported into plants, entering different metabolic pathways by substituting the metals present at the binding site or binding with different proteins, making them inactive. Cd changes the redox environment, implying metabolic disturbances. The generation of high concentrations of ROS is the source of plant oxidative stress in Cd-rich environments [28]. High ROS concentrations are detrimental to membrane structure and lipid and protein function; the extent of injury depends on the species and stress intensity [13,27].

Our study showed significant modulation of ROS-scavenging activities in lentil roots in response to Cd stress. Roots are the prime organ responsible for Cd uptake in plants. Cd is transported to shoots and other tissues after sequestration into vacuoles, translocation into xylem and phloem, and dilution through shoot growth. Root ultrastructure and antioxidative activities are important for stress tolerance in pea under Cd stress [29].

The stress-induced injury was apparent in both genotypes as H_2_O_2_ and MDA contents increased in both Cd treatments, though JL3 had higher susceptibility to Cd stress than L4717 (Figure 1A,B). Similar findings have been reported in mung bean [4], chickpea [13], and faba bean [26]. JL3 had greater oxidative damage than L4717, evident by greater accumulation of H_2_O_2_ and lipid peroxidation products in the presence of Cd. While H_2_O_2_ is a stable molecule with a comparatively long half-life relative to other ROS, it can trigger damage if it moves across the membrane. CAT and POX activities are critical for catalyzing the dissociation of H_2_O_2_, with POX having a higher affinity for H_2_O_2_ than CAT. L4717 had higher CAT and POX activities than JL3 in the presence of 100 µM and 200 µM Cd (Figure 1C,D), indicating that L4717 is more efficient at H_2_O_2_ degradation than JL3. Elevated enzymatic activities may be associated with excessive H_2_O_2_ generation as observed in Cd-stressed plants [14]. Genes encoding CAT were upregulated in plants exposed to heavy metal stress [30]. However, the increase in POX and CAT activities under Cd stress was insufficient to remove H_2_O_2_, and therefore, it accumulated in L4717 and JL3.

SOD is a key enzyme, which dismutates superoxide radicals into H_2_O_2_ and O_2_. SOD activity was significantly upregulated in L4717 in the presence of Cd, but declined in JL3 (Figure 1E). Differences in SOD activity demonstrate the dearth of antioxidant capacity in JL3 to cope with Cd-induced oxidative stress. Hence, MDA accumulation increased in JL3 under stress (Figure 1B). The higher SOD activity in L4717 was ascribed to its high seed Fe concentration (65 ppm) compared to JL3 (45 ppm). The low availability of Fe chelates in JL3 leads to a low level of SOD activity, as reported for barley [31]. GR is the enzyme responsible for reducing oxidized glutathione in a reduced form, and NADPH is required for GR activity. GR is responsible for maintaining reduced glutathione levels by H_2_O_2_ elimination. L4717 had significantly higher GR activity than JL3 under both Cd levels (Figure 1F), which enhanced its stress tolerance and ability to eliminate H_2_O_2_. Cd stress-mediated increases in GR activity were also reported in mung bean [4] and chickpea [13].

Heavy metal tolerance improved in chickpea as H_2_O_2_ content and cell membrane injury declined with exogenous Ca and K treatments [13]. Fe supplementation improved the oxidative response in mung bean by modulating enzyme activities in Cd-enriched conditions [4]. Growth of low seed Fe genotype JL3 declined more in both Cd treatments due to notable oxidative injuries and high Cd uptake, despite increased antioxidant activities in both the genotypes (Figure 1, Figure 2 and Figure 3). This study showed that lentil genotypes acclimatized to Cd stress by exerting strong antioxidant activities, with POX being the key player in H_2_O_2_ detoxification. The higher antioxidant capacity of L4717 suggests that Fe-rich genotypes can alleviate Cd toxicity more effectively than genotypes with low seed Fe concentration. Fe supplementation improved Cd tolerance in mung bean [4] and rice [32] by maintaining chlorophyll content and modulating redox status and Cd uptake. It was proposed that Fe-rich conditions ensure Fe availability, a ligand for different antioxidant enzymes, and a binding force for forming multi-protein complexes [12].

All measured growth traits (TL, SL, RL, FW, and DW) decreased significantly in lentils exposed to Cd stress (Table 2), and more so in JL3 than L4717 (Figure 2). Plant weight and TL had negative correlations with oxidative injury indicators under Cd stress (Figure 4). JL3 had significant injuries and stunted growth, indicating its high susceptibility to Cd stress. JL3 also had higher Cd uptake than L4717 under Cd stress (Figure 3). Cd content had negative correlations with all measured growth traits, but Fe-rich L4717 sustained growth better than JL3. As reported in mung bean and rice, Fe supplementation was associated with less Cd accumulation under stress through modulated transporter activities [4,12]. We also observed that Fe-biofortified L4717 accumulated less Cd than JL3. Fe is a co-factor of antioxidase, which protects against Cd toxicity [33]. No significant correlations were recorded between growth traits and enzymatic activities in lentils, except for a positive correlation between FW and POX activity, indicating that POX is the key enzyme contributing to Cd tolerance in lentils (Figure 4). In mung bean and chickpea, GR activity was highly correlated with Cd stress tolerance [4,12,13].

In summary, this study revealed that Cd stress significantly affects lentil growth by oxidative damage. Cd stress tolerance in biofortified L4717 was attributed to strong antioxidant potential and improved growth in toxic environments due to high seed Fe content. We suggest that the biofortification of lentil varieties may help reduce malnutrition and improve Cd tolerance. The role of foliar application of Fe should be studied in lentil varieties to improve Cd tolerance. Using high Fe content as a surrogate tool for Cd tolerance in lentils and other legume crops needs to be explored.

## Figures and Tables

**Table 1:** Analysis of variance (ANOVA) F values for biochemical traits between lentil genotypes, Cd treatments, and their interaction.

Source of Variation	HP	MDA	POX	CAT	SOD	GR
Treatment	728.59 **	111.71 **	587.71 **	47.55 **	238.04 **	28.63 **
Genotype	32.55 **	201.35 **	374.29 **	30.75 **	133.79 **	32.19 **
Treatment × Genotype	77.26 **	14.18 **	67.68 **	22.47 **	69.76 **	10.27 **

* Significant at *p* < 0.05, ** significant at *p* < 0.01; HP: hydrogen peroxide; MDA: malondialdehyde; POX: peroxidase; CAT: catalase; SOD: superoxide dismutase; GR: glutathione reductase.

**Table 2:** ANOVA of F values for growth traits and Cd uptake between lentil genotypes, Cd treatments, and their interaction.

Source of Variation	TL	RL	SL	FW	DW	CdC
Treatment	94.93 **	9.21 **	58.86 **	368.26 **	45.80 **	3483.91 **
Genotype	144.04 **	84.88 **	88.54 **	98.37 **	64.80 **	871.36 **
Treatment × Genotype	21.32 **	3.85 *	2.52 *	3.86 *	2.90 *	430.83 **

* Significant at *p* < 0.05, ** significant at *p* < 0.01, TL: total length, RL: root length, SL: shoot length, FW: fresh weight, DW: dry weight, CdC: Cd content.
